# Inhibition of nuclear factor-κB signal by pyrrolidine dithiocarbamate alleviates lipopolysaccharide-induced acute lung injury

**DOI:** 10.18632/oncotarget.17624

**Published:** 2017-05-04

**Authors:** Hongfu Yang, Rongqing Sun, Ning Ma, Qilong Liu, Xiaoge Sun, Panpan Zi, Junsheng Wang, Ke Chao, Lei Yu

**Affiliations:** ^1^ Critical Care Medical Department, The First Affiliated Hospital of Zhengzhou University, Zhengzhou 450000, P.R. China

**Keywords:** NF-κB, acute lung injury, inflammation, oxidative stress, mitochondrial function

## Abstract

This study mainly studied the effect of inhibition of nuclear factor-κB (NF-κB) signal by pyrrolidine dithiocarbamate (PDTC) on lipopolysaccharide (LPS)-induced inflammatory response, oxidative stress, and mitochondrial dysfunction in a murine acute lung injury model. The results showed that LPS exposure activated NF-κB and its upstream proteins and caused lung inflammation, oxidative stress, and mitochondrial dysfunction in mice. While inhibition of NF-κB by PDTC adminstration alleviated LPS-induced generation of lymphocytes, IL-1β, and TNF-α. Malondialdehyde, a common oxidative product, was markedly reduced after PDTC treatment in LPS-challenged mice. Furthermore, PDTC alleviated LPS-induced mitochondrial dysfunction via improving ATP synthesis and uncoupling protein 2 expression. In conclusion, inhibition of NF-κB by PDTC alleviated LPS-induced acute lung injury via maintaining inflammatory status, oxidative balance, and mitochondrial function in mice.

## INTRODUCTION

Acute lung injury is a major causes of acute respiratory failure characterized by oxidative stress, inflammatory response, neutrophil accumulation, diffuse endothelium and epithelial damage, air-blood barrier disruption, and the subsequent infiltration of peripheral inflammatory cells into lung tissues [1, 2]. Although a large number of studies have focused on the pathogenesis and therapies, very few therapies for acute respiratory failure have been shown to be effective. Therefore, investigations on the molecular mechanisms underlying the progression of acute respiratory failure may have a significant impact on the systematic treatment of this disease.

Nuclear factor-κB (NF-κB), a transcription factor of DNA, cytokine, and cell survival, has been widely demonstrated to involve in cellular responses to various stress, such as cytokines, free radicals, heavy metals, and bacterial or viral antigens. Overexpression or inappropriate activation of NF-κB implicated in a number of pathological mechanisms of diseases ranging from inflammation to cancer. In the acute lung injury, NF-κB has been widely served as the therapeutic target to alleviate inflammation. For example, acteoside, tylvalosin, and emodin were demonstrated to inhibit NF-κB signal, which further alleviated inflammatory response in acute lung injury models [3–5]. Small interfering RNA (siRNA) against NF-κB also confirmed the beneficial effects of NF-κB inhibition on inflammatory response, including acute lung injury model [6]. Thus, inhibition of the NF-κB pathway considers as a potential strategy for the therapeutic target of this crucial transcription factor of acute lung injury. Pyrrolidine dithiocarbamate (PDTC) is a thiol compound and has been considered as an effective inhibitor of NF-kB [7–9]. Thus, we used PDTC to inhibit NF-κB pathway to investigate the protective effects of NF-κB inactivation by PDTC on lipopolysaccharide (LPS)-induced acute lung injury in mice.

## RESULTS

### NF-κB activity

**Figure 1:**
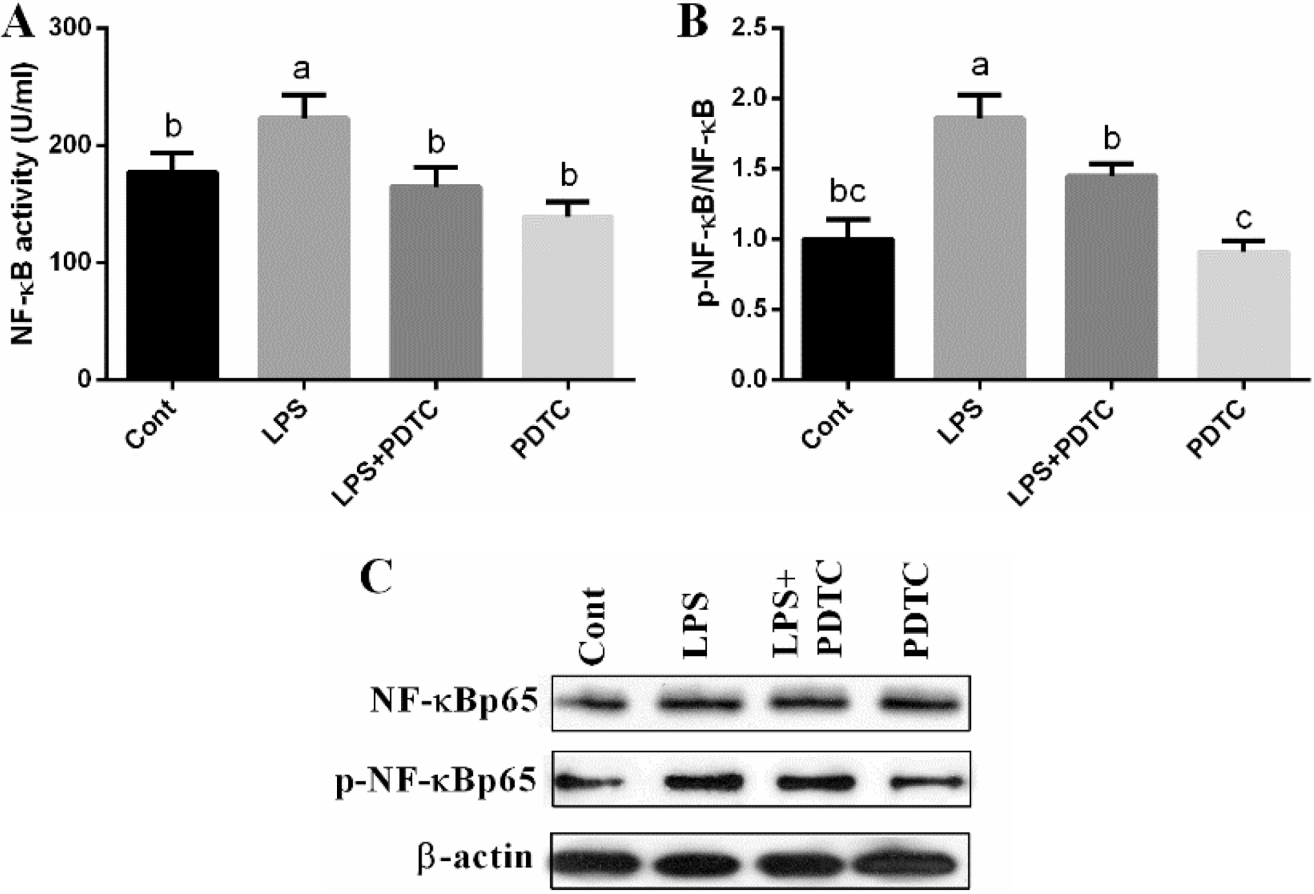
Effects of LPS and PDTC of NF-κB signal in the lung via ELISA kit and western blot Data are expressed as the mean ± standard error of the mean. Values in the same row with different superscripts are significant (*P* < 0.05).

NF-κB activity was tested using ELISA kit and the results showed that LPS activated NF-κB signal (*p <* 0.05), suggesting that NF-κB involved in LPS-induced acute lung injury. Meanwhile, PDTC exposure markedly inhibited NF-κB activity (*p <* 0.05), which might serve as a protective mechanism on LPS-induced acute lung injury. The result was further confirmed by western blotting analysis, which showed that PDTC treatment inhibited LPS-induced phosphorylation of NF-κBp65 (*p <* 0.05) (Figure 1B and 1C).

### TLRs/Myd88

**Figure 2:**
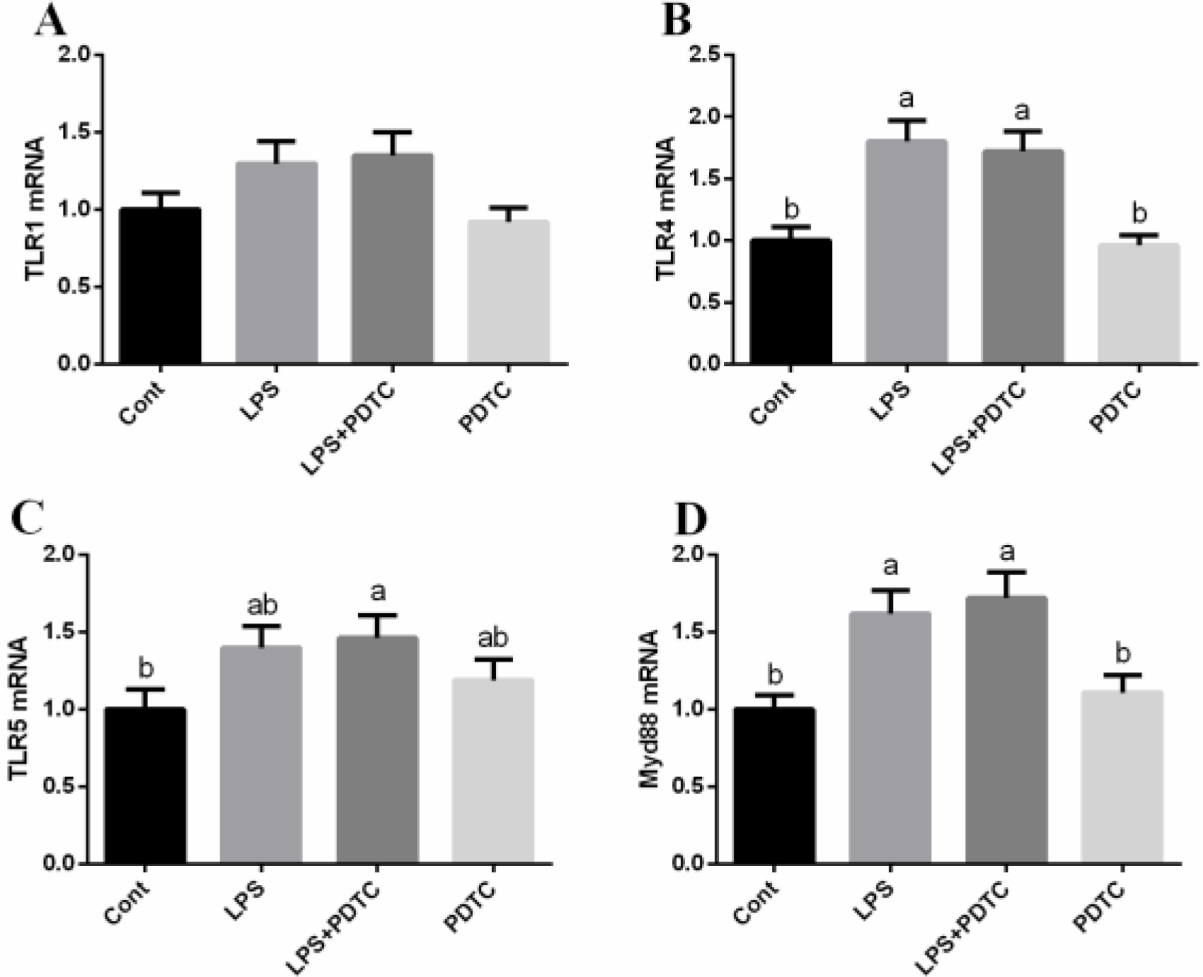
Effects of NF-κB inhibition on TLRs/Myd88 in the lung via RT-PCR Data are expressed as the mean ± standard error of the mean. Values in the same row with different superscripts are significant (*P* < 0.05).

TLRs/Myd88 serves as the upstream of NF-κB signaling pathway, thus we further determined TLR1, TLR4, TLR5, and Myd88 expressions in the lung after LPS treatment (Figure 2). We found that LPS markedly upregulated TLR4 and Myd88 expression (*p <* 0.05), while PDTC failed to influence the TLRs/Myd88 signal.

### PDTC alleviates LPS-induced inflammatory cells infiltration and inflammatory response

**Figure 3:**
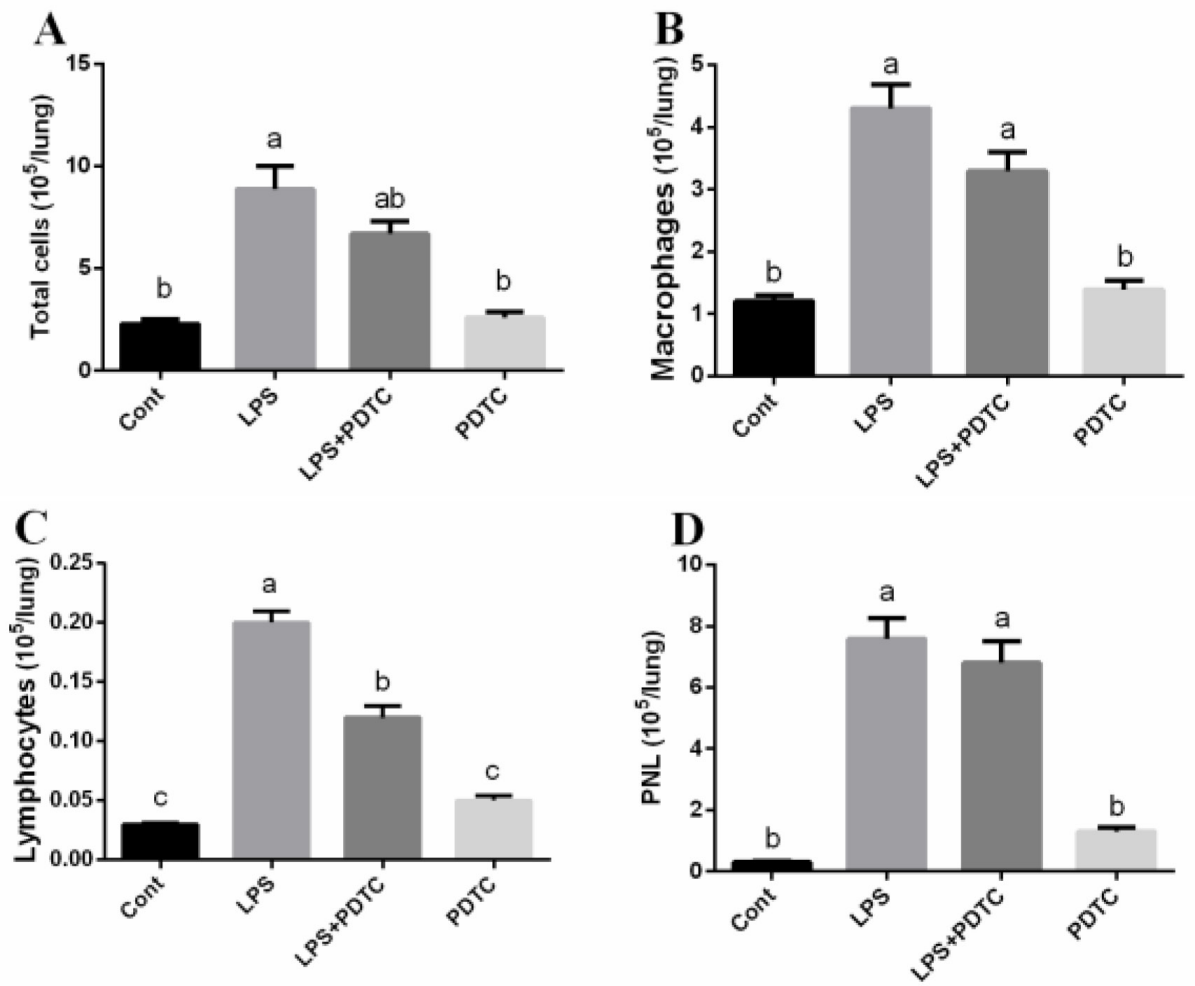
PDTC alleviates LPS-induced inflammatory cells infiltration in the lung Data are expressed as the mean ± standard error of the mean. Values in the same row with different superscripts are significant (*P* < 0.05).

BAL was used to test the inflammatory cells, including macrophages, lymphocytes, and PNL (Figure 3). Total cells, macrophages, lymphocytes, and PNL were markedly higher in LPS-changed group compared with that in the control group (*p <* 0.05). PDTC tended to reduce total cells and macrophages in BAL fluid, but the difference was insignificant (*p >* 0.05). Lymphocytes was significantly decreased in LPS+PDTC group compared with the LPS group (*p <* 0.05). We further tested immunoglobulins (IgA, IgG, and IgM) in the BAL fluid and found that LPS markedly reduced IgG and IgM abundances (*p <* 0.05) (Table 1), while PDTC failed to influence immunoglobulins secretion in the lung (*p >* 0.05).

**Table 1:** Effects of LPS and PDTC on lung immunoglobulins (g/L)

Item	Cont	LPS	LPS+PDTC	PDTC
IgA	1.65 ± 0.16	1.93 ± 0.15	1.47 ± 0.15	1.75 ± 0.27
IgG	8.46 ± 0.57^a^	6.53 ± 0.42^b^	7.07 ± 0.23^b^	8.94 ± 0.59^a^
IgM	0.37 ± 0.05^a^	0.31 ± 0.02^b^	0.34 ± 0.06^ab^	0.38 ± 0.06^a^

**Figure 4:**
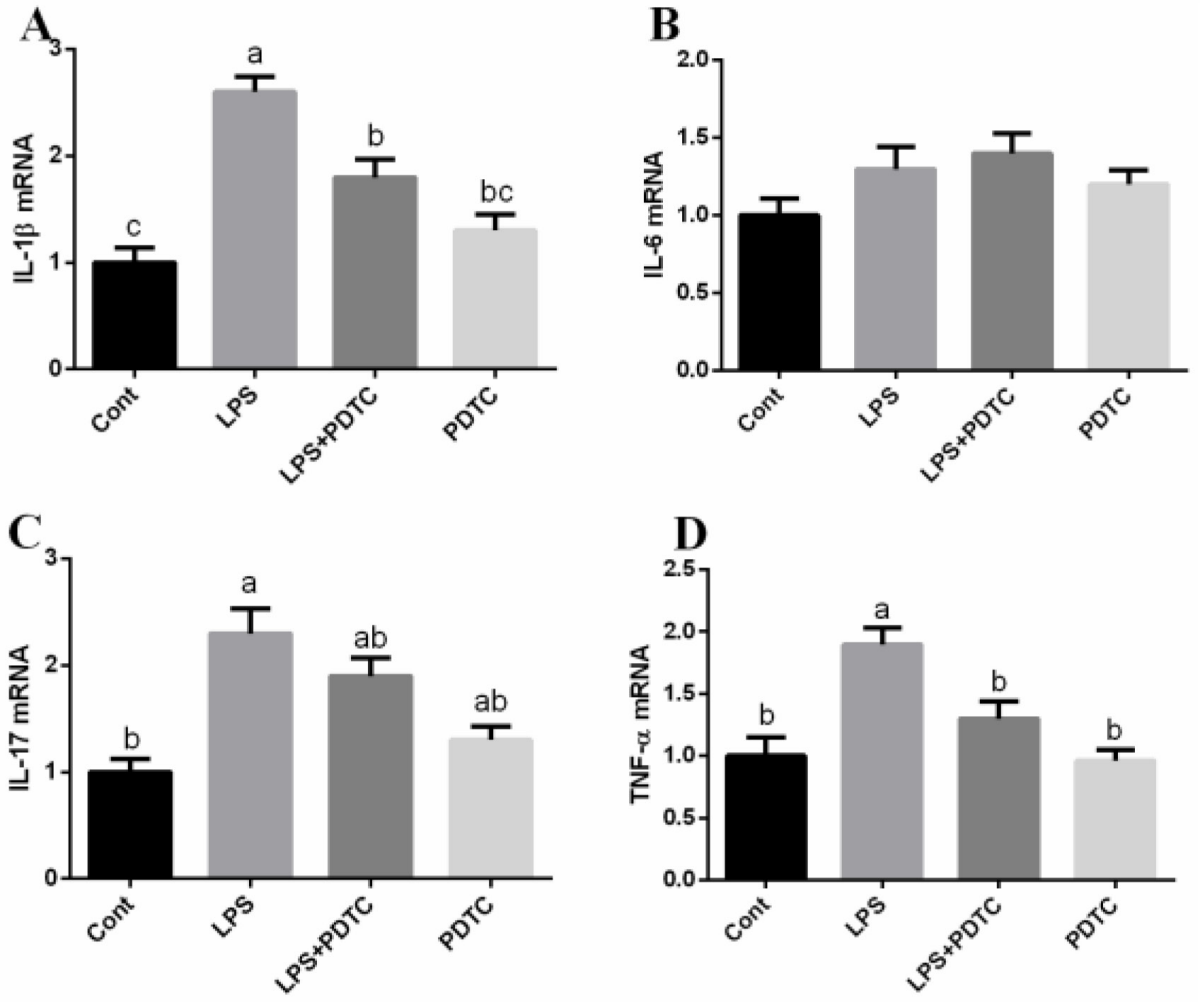
PDTC alleviates LPS-induced inflammatory response in mice IL-1β, IL-6, IL-17, and TNF-α were determined by RT-PCR. Data are expressed as the mean ± standard error of the mean. Values in the same row with different superscripts are significant (*P* < 0.05).

Expressions of IL-1β, IL-6, IL-17, and TNF-α in the lung were further tested via RT-PCR (Figure 4). The results showed that LPS exposure upregulated IL-1β, IL-17, and TNF-α expression (*p <* 0.05) and PDTC alleviated LPS-induced generation of L-1β and TNF-α (*p <* 0.05).

### PDTC alleviates LPS-induced oxidative stress in mice

Total antioxidant capacity (T-AOC) and Malondialdehyde (MDA) were determined to evaluate the oxidative stress after LPS exposure in mice Table 2. The results showed that LPS treatment markedly induced oxidative stress in the lung evidenced by the enhanced MDA level (*p <* 0.05), while PDTC reduced MDA production compared with the LPS group (*p <* 0.05), indicating an antioxidant effect of PDTC on LPS-induced acute lung injury.

**Table 2:** PDTC alleviates LPS-induced oxidative stress in mice

Item	Cont	LPS	LPS+PDTC	PDTC
T-AOC U/gprot	0.43 ± 0.06	0.31 ± 0.07	0.35 ± 0.08	0.49 ± 0.09
MDA uM/mgprot	12.17 ± 1.36bc	17.54 ± 1.72a	13.29 ± 1.13b	10.27 ± 1.25c

Data are expressed as the mean ± standard error of the mean. Values in the same row with different superscripts are significant (*P* < 0.05).

**Figure 5:**
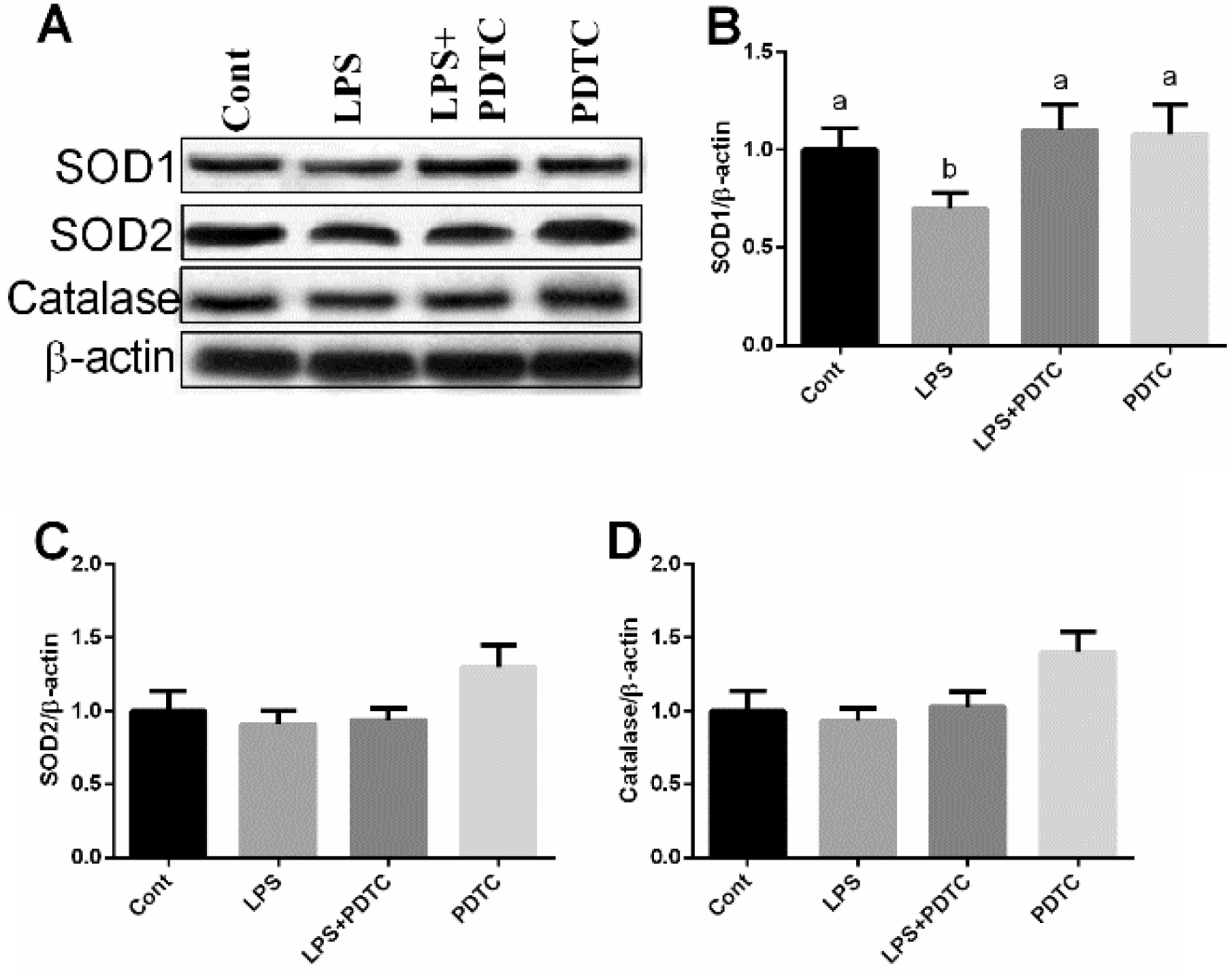
Effects of LPS and PDTC on SOD1, SOD2, and catalase expressions in the lung via western blot Data are expressed as the mean ± standard error of the mean. Values in the same row with different superscripts are significant (*P* < 0.05).

Expressions of superoxide dismutase 1 (SOD1), SOD2, and catalase in the lung after LPS exposure were further determined via western blot (Figure 5). The results showed that LPS inhibited SOD1 expression and PDTC markedly enhanced SOD1 abundance in the lung (*p <* 0.05). Although PDTC tended to upregulated SOD2 and catalase expressions, the differences were insignificant (*p >* 0.05).

### PDTC alleviates LPS-induced mitochondrial dysfunction in mice

**Figure 6:**
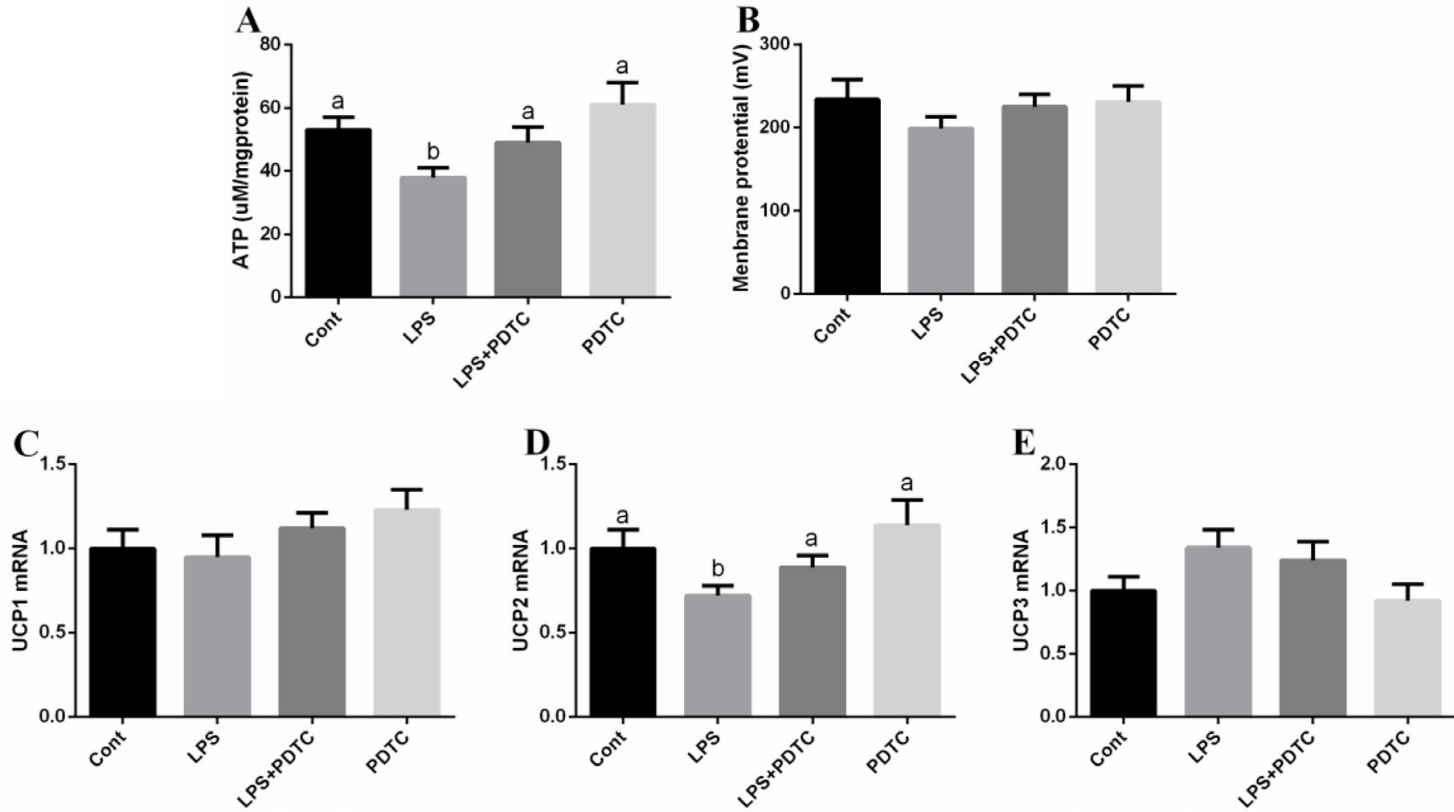
Mitochondrial function in the lung after LPS and PDTC treatment UCP1-3 were determined by RT-PCR. Data are expressed as the mean ± standard error of the mean. Values in the same row with different superscripts are significant (*P* < 0.05).

Mitochondrial function (ATP synthesis and membrane potential) was evaluated (Figure 6) and the results showed that LPS markedly induced mitochondrial dysfunction via inhibiting ATP synthesis (*p <* 0.05), while PDTC alleviated the mitochondrial dysfunction (*p <* 0.05).

Uncoupling proteins (UCPs) contribute to oxidative phosphorylation from ATP synthesis and mitochondrial proton leak. In this study, we found that LPS inhibited UCP2 expression in the lung (*p <* 0.05) (Figure 5), which further confirmed the mitochondrial dysfunction after LPS exposure. Meanwhile, PDTC alleviated the inhibitory effect of LPS on UCP2 expression (*p <* 0.05).

## DISCUSSION

NF-κB involves in various inflammatory diseses and mediates cytokines expression [10, 11]. In this study, we found that LPS significantly increased NF-κB activity and its upstream proteins (TLR4/Myd88), suggesting that TLR4/Myd88/NF-κB involved in LPS-induced inflammation and oxidative stress in mice. PDTC has been widely served as the inhibtor of NF-κB and exhibits antioxidant function in various models [12, 13]. The present study used PDTC to inhibit NF-κB signal in LPS-induced acute lung injury and showed that inhibition of NF-κB alleviated LPS-induced inflammation, oxidative stress, and mitochondrial dysfunction in mice.

Inflammation plays a critical role in the progression of acute lung injury [14]. The current results indicated a marked inflammatory response in LPS-challenged mice evidenced by the increased inflammatory cells (macrophages, lymphocytes, and PNL) and over-expressions of IL-1β, IL-17, and TNF-α and the reduced immunoglobulins (IgG and IgM) in the lung, while inhibition of NF-κB by PDTC treatment alleviated LPS-induced generation of lymphocytes, L-1β, and TNF-α. Similarly, Li et al. reported the effects of NF-κB inhibitor PDTC on a herpetic stromal keratitis mouse model and showed that 10 mg/mL PDTC treatment markedly decreased IL-1β and IL-4 expressions [12]. Although we failed to investigated the morphology in the lung after LPS exposure, histologic analysis from a murine model of titanium particulate-induced inflammation showed that PDTC alleviated lung injury and decreased cellular infiltration and the release of inflammatory cytokines (IL-1β and TNF-α) in the lavage fluid [15].

Oxidative stress has been indicated to contribute to the progression of acute lung injury [16–19]. Under oxidative stress, oxidative balance was disturbed with production of oxidative products [20], such as MDA. In this study, we found that MDA level in the lung was enhanced in LPS-induced acute lung injury in mice, while inhibition of NF-κB markedly alleviated oxidative injury in LPS-challenged mice. NF-κB also serves as a redox-sensitive factor and oxidative stress plays an important role in the activation of NF-κB signal [21, 22]. Meanwhile, provious reports also confirmed an antioxidant function of PDTC [23, 24]. For example, PDTC adminstration inhibited superoxide anion-induced NF-κB activation, inflammation, and oxidative stress in the paw and spinal cord, which further alleviated mechanical hyperalgesia, thermal hyperalgesia and inflammatory response in peripheral foci [23]. Similarly, we also noticed that PDTC enhanced SOD1 expression, suggesting that PDTC improve antioxidant balance in LPS-induced acute lung injury in mice.

LPS-induced acute lung injury has been demonstrated to be characterized by abnormal mitochondrial structures and dysfunctions [25]. In this study, LPS exposure inhibited ATP synthesis and expression of UCP2, a family of mitochondrial anion carrier proteins, contributing to oxidative phosphorylation from ATP synthesis and mitochondrial proton leak [26, 27]. Varela et al. reported that mitochondria controlled by UCP2 determine hypoxia-induced synaptic remodeling in the cortex and hippocampus [28]. Meanwhile, UCP2 also regulates mitochondrial glucose metabolism via transporting C4 metabolites out of mitochondria [29]. Mitochondrial dysfunction may be a main cause for acute lung injury, thus improving mitochondrial function may serve as a potential treating acute lung injury [25]. Inhibition of NF-κB by PDTC alleviated LPS-induced mitochondrial dysfunction in this study, suggesting a protective role of PDTC in LPS-induced acute lung injury.

## MATERIALS AND METHODS

### Animal model and groups

This study was approved by the animal welfare committee of The First Affiliated Hospital of Zhengzhou University. 40 8-week-old female Balb/c mice were randomly assigned into 4 groups (*n* = 10): a control group, a LPS-challenged group, a PDTC group, and a PDTC plus LPS group. LPS was used to induce acute lung injury via intraperitoneal injection of 15 mg/kg LPS (Sigma, St. Louis, MO, USA). PDTC (Sigma-Aldrich Co., USA), dissolved in distilled water, was administered intraperitoneally to mice at dose levels of 50 mg/kg 1 hour before LPS treatment.

### Inflammatory cell

After 24 h, mice were sacrificed and the lungs were lavaged twice with 0.8 mL sterile saline each time to obtain bronchoalveolar lavage fluid (BAL). BAL was performed by cannulating the trachea with a 20-gauge needle and infusing the lungs 2 times with 1 ml of physiological buffered saline (PBS) containing 0.1% bovine serum albumin (BSA). BAL fluid was centrifuged (150 × g) for 10 min at 4 °C. The cells obtained were resuspended in 500 μL of PBS containing 0.1% BSA and used to measure the total cell count and macrophages, lymphocytes, and polymorph nuclear leukocytes (PNL).

### NF-κB activity

Lung samples were homogenized (1 g tissue in 9 mL saline) and then centrifuged at 3,000 × g for 10 min under 4°C. The supernatants were used for determining NF-κB activity via an ELISA kit (Shanghai Yaji Bio. Tech., China).

### Oxidative stress

Lung T-AOC activity was measured using spectrophotometric kits (Nanjing Jiangcheng Biotechnology Institute, China). MDA was measured using a thiobarbituric acid reactive substances assay kit according to the manufacturer's instructions (Nanjing Jiangcheng Biotechnology Institute, China).

### Mitochondrial function

Mitochondria from mouse lungs were isolated and ATP synthesis was measured with a luciferase/luciferin-based approach. The mitochondrial membrane potential (ΔΨm) was estimated using Rhodamine (Sigma) according to previous report.

### Real-time PCR

One piece of lung were harvested and stored at −80°C. Total RNA of these tissues was isolated with TRIZOL regent (Invitrogen, USA) and reverse transcribed into the first strand (cDNA) using DNase I, oligo (dT) 20 and Superscript II reverse transcriptase (Invitrogen, USA). The reverse transcription was conducted at 37°C for 15 min, 95°C 5 sec. Primers were designed with Primer 5.0 according to the gene sequence of mouse to produce an amplification product (Table 3). β-actin was chosen as the house-keeping gene to normalize target gene levels. The PCR cycling condition was 36 cycles at 94°C for 40 sec, 60°C for 30 sec and 72°C for 35 sec. The relative expression was expressed as a ratio of the target gene to the control gene using the formula 2^-(ΔΔCt)^, where ΔΔCt=(Ct_Target_-Ct_β-actin_)_treatment_-(Ct_Target_-Ct_β-actin_)_control_. Relative expression was normalized and expressed as a ratio to the expression in the control group.

**Table 3:** Primers used in this study

Genes	No.	Nucleotide sequence of primers (5′–3′)	bp
β-Actin	NM_007393.5	F: CCACCATGTACCCAGGCATT R: AGGGTGTAAAACGCAGCTCA	253
IL-1β	NM_008361.4	F: TGCCACCTTTTGACAGTGATG R: AAGGTCCACGGGAAAGACAC	220
IL-6	NM_031168.2	F: CCCCAATTTCCAATGCTCTCC R: CGCACTAGGTTTGCCGAGTA	141
IL-17	NM_010552.3	F: GCTGACCCCTAAGAAACCCC R: GAAGCAGTTTGGGACCCCTT	162
TNF-α	NM_013693.3	F: ATGGCCTCCCTCTCATCAGT R:TTTGCTACGACGTGGGCTAC	*97*
TLR1	*NM_001276445.1*	F: ACGGGTAAGGTTGTCTTGACG R: TTCCGCTCTCTTCATGCCTC	*108*
TLR4	*NM_021297.3*	F: CCATGCATTTGGCCTTAGCC R: AGAGCACTGAACCTCCTTGC	*74*
TLR5	NM_016928.3	F: GAATCCCGCTTGGGAGAACA R: TTCCAAGCGTAGGTGCTCTG	159
Myd88	NM_010851.2	F: GCTGGCAGGAGACTTAAGGG R: TCCGAGGGTTCAAGAACAGC	201

F: forward; R: reverse.

### Western blot

Proteins of lung were extracted with using protein extraction reagents (Thermo Fisher Scientific Inc., USA) and the concentration was tested using BCA protein assay (Sigma-Aldrich, USA). Proteins (30 μg) were separated by SDS–polyacrylamide gel electrophoresis and electrophoretically transferred to a polyvinylidene difluoride (PVDF) membrane (BioRad, Hercules, CA, USA). Membranes were blocked and then incubated with the following primary antibodies: anti-NF-kBp65 (ab16502), anti-NF-kBp65 (phospho S536) antibody (ab86299), anti-superoxide dismutase 1 antibody [SOD1] (ab20926), anti-SOD2/MnSOD antibody (ab13533), anti-Catalase antibody (ab16731), and anti-beta Actin antibody (ab8227). After primary antibody incubation, membranes were washed, incubated with alkaline phosphatase-conjugated anti-mouse or anti-rabbit IgG antibodies (Promega, Madison, WI, USA), and quantified and digitally analyzed using the image J program (NIH).

### Statistical analysis

All data were analyzed by SPSS 17.0 software. Difference was tested by Ducan's multiple comparison test. Data are expressed as the mean ± SEN. Values in the same row with different superscripts are significant (*P* < 0.05).
